# Update: Influenza Activity — United States, September 29–December 7, 2013

**Published:** 2013-12-20

**Authors:** World Health Organization Collaborating Center for Surveillance, Epidemiology, and Control of Influenza, Atlanta, Georgia, Lynnette Brammer, Scott Epperson, Lenee Blanton, Krista Kniss, Desiree Mustaquim, Craig Steffens, Rosaline Dhara, Michelle Leon, Alejandro Perez, Sandra Chaves, Jackie Katz, Teresa Wallis, Julie Villanueva, Xiyan Xu, Anwar Isa Abd Elal, Larisa Gubareva, Lyn Finelli, Joseph Bresee, Nancy Cox, Alexander Millman

**Affiliations:** Influenza Div, National Center for Immunization and Respiratory Diseases; EIS Officer, CDC

CDC collects, compiles, and analyzes data on influenza activity year-round in the United States ( http://www.cdc.gov/flu/weekly/fluactivitysurv.htm ). The influenza season generally begins in the fall and continues through the winter and spring months; however, the timing and severity of circulating influenza viruses can vary by geographic location and season. Influenza activity in the United States continued to increase from mid-November through the beginning of December. This report summarizes U.S. influenza activity* during September 29–December 7, 2013.†

## Viral Surveillance

**FIGURE 1:**
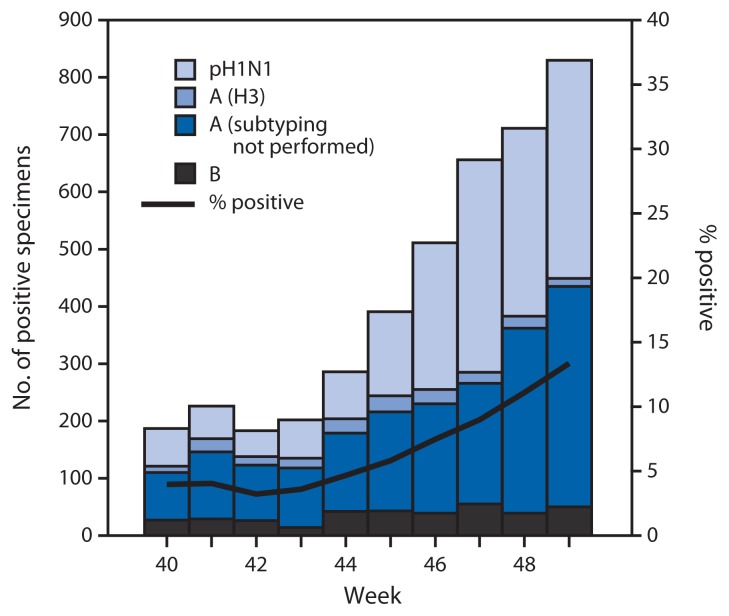
Number^*^ and percentage of respiratory specimens testing positive for influenza reported by World Health Organization and National Respiratory and Enteric Virus Surveillance System collaborating laboratories in the United States, by type, subtype, and week — United States, September 29–December 7, 2013^†^ ^*^ N = 4,183. ^†^ Data reported as of December 13, 2013.

During September 29–December 7, 2013, approximately 140 World Health Organization (WHO) and National Respiratory and Enteric Virus Surveillance System collaborating laboratories in the United States tested 61,261 respiratory specimens for influenza viruses; 4,183 (6.8%) were positive (Figure 1). Of these, 3,819 (91.3%) were influenza A viruses, and 364 (8.7%) were influenza B viruses. Of the 3,819 influenza A viruses, 1,998 (52.3%) were subtyped; 198 (10%) of these were influenza A (H3) viruses, and 1,800 (90%) were influenza A (H1N1)pdm09 (pH1N1) viruses. Since September 29, 2013, influenza-positive tests have been reported from 48 states, the District of Columbia, and Puerto Rico, representing all 10 U.S. Department of Health and Human Services (HHS) regions.§ Thus far, influenza A viruses have predominated nationally and in all 10 HHS regions.

## Novel Influenza A Viruses

One infection with an influenza A (H3N2) variant virus (H3N2v) was reported to CDC from Iowa during the week ending October 12, 2013 (week 41). Contact between the patient and swine in the week preceding illness was reported. The patient was mildly ill and fully recovered; no further cases have been identified among contacts of the patient. This is the first H3N2v infection reported for the 2013–14 influenza season.

## Antigenic Characterization

WHO collaborating laboratories in the United States are requested to submit a subset of their influenza-positive respiratory specimens to CDC for further antigenic characterization (1). CDC has antigenically characterized 221 influenza viruses collected by U.S. laboratories during the 2013–14 season, including 184 pH1N1 viruses, 31 influenza A (H3N2) viruses, and six influenza B viruses. All pH1N1 and influenza A (H3N2) viruses were antigenically like the 2013–14 Northern Hemisphere influenza A vaccine components (A/California/7/2009-like [H1N1] and A/Texas/50/2012-like [H3N2]). Two (33%) of the influenza B viruses tested belong to the B/Yamagata lineage and were characterized as B/Massachusetts/2/2012-like, which is included as an influenza B component in the 2013–14 Northern Hemisphere trivalent and quadrivalent influenza vaccines. The remaining four (67%) influenza B viruses tested belong to the B/Victoria lineage and were characterized as B/Brisbane/60/2008-like, which is included as an influenza B component in the 2013–14 Northern Hemisphere quadrivalent influenza vaccine.

## Antiviral Resistance of Influenza Viruses

Testing of pH1N1, influenza A (H3N2), and influenza B virus isolates for resistance to neuraminidase inhibitors (oseltamivir and zanamivir) is performed at CDC using a functional assay. Additional pH1N1 and influenza A (H3N2) clinical samples are tested for mutations of the virus known to confer oseltamivir resistance. Since October 1, 2013, a total of 463 influenza viruses have been tested for antiviral resistance, including 395 pH1N1 viruses, 55 influenza A (H3N2) viruses, and 13 influenza B viruses. Of the 395 pH1N1viruses tested, seven (1.8%) were resistant to oseltamivir. Of the 273 pH1N1 viruses tested, all (including the seven oseltamivir-resistant viruses) were sensitive to zanamivir. Of the 55 influenza A (H3N2) viruses and 13 influenza B viruses tested, all were sensitive to both oseltamivir and zanamivir.

## Outpatient Illness Surveillance

**FIGURE 2:**
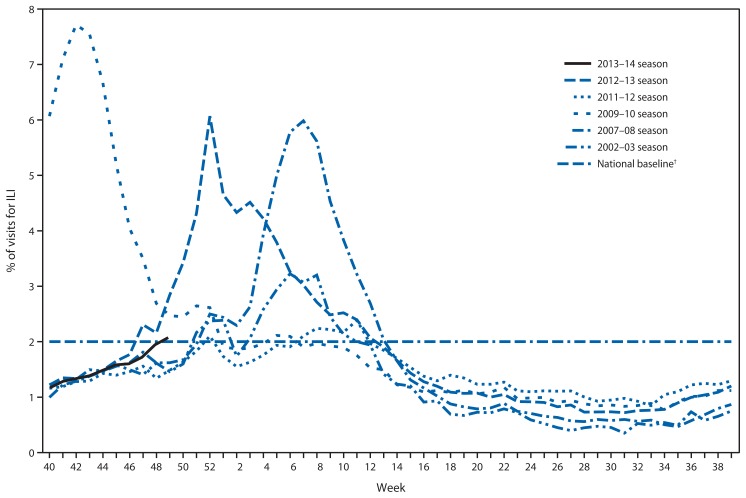
Percentage of visits for influenza-like illness (ILI)^*^ reported to CDC, by surveillance week — Outpatient Influenza-Like Illness Surveillance Network, United States, September 29–December 7, 2013, and selected previous influenza seasons ^*^ Defined as a temperature of ≥100°F (≥37.8°C), oral or equivalent, and cough and/or sore throat, without a known cause other than influenza. ^†^ The national baseline is the mean percentage of visits for ILI during noninfluenza weeks for the previous three seasons plus two standard deviations. Noninfluenza weeks are defined as periods of ≥2 consecutive weeks in which each week accounted for <2% of the season’s total number of specimens that tested positive for influenza. National and regional percentages of patient visits for ILI are weighted on the basis of state population. Use of the national baseline for regional data is not appropriate.

Since September 29, 2013, the weekly percentage of outpatient visits for influenza-like illness (ILI)¶ reported by approximately 1,800 U.S. Outpatient ILI Surveillance Network (ILINet) providers in 50 states, New York City, Chicago, the U.S. Virgin Islands, Puerto Rico, and the District of Columbia, which comprise ILINet, has ranged from 1.2% to 2.1% and was at or above the national baseline** of 2.0% during the weeks ending November 30, 2013, and December 7, 2013 (weeks 48 and 49) (Figure 2). Peak weekly percentages of outpatient visits for ILI ranged from 2.4% to 7.6% from the 1997–98 through 2012–13 seasons, excluding the 2009 pandemic. For the week ending December 7, 2013 (week 49), three regions (HHS regions 4, 6, and 8) reported ILI activity above their region-specific baseline levels. This is the fourth week this season during which one or more region-specific baselines were exceeded. Data collected in ILINet are used to produce a measure of ILI activity†† by jurisdiction. During week 49, four states (Alabama, Louisiana, Mississippi, and Texas) experienced high ILI activity, no jurisdictions experienced moderate ILI activity, and five states (Arkansas, Colorado, Oklahoma, South Carolina, and Utah) and New York City experienced low ILI activity. Forty-one states experienced minimal ILI activity, and data were insufficient to calculate an ILI activity level for the District of Columbia.

## Geographic Spread of Influenza Activity

For the week ending December 7, 2013 (week 49), no jurisdictions reported the geographic spread of influenza§§ as widespread, 14 states (Alabama, Arkansas, Colorado, Florida, Kentucky, Louisiana, Maine, Maryland, Mississippi, North Carolina, Oklahoma, South Carolina, Texas, and Utah) reported regional spread, and 18 states (Alaska, Arizona, Connecticut, Georgia, Illinois, Kansas, Massachusetts, Michigan, Minnesota, Nebraska, Nevada, New Jersey, New York, Ohio, Oregon, Tennessee, Virginia, and Wyoming) reported local spread. Sporadic influenza activity was reported by the District of Columbia, Guam, Puerto Rico, and 16 states. No influenza activity was reported by the U.S. Virgin Islands and two states (New Hampshire and Vermont).

## Influenza-Associated Hospitalizations

CDC monitors hospitalizations associated with laboratory-confirmed influenza in adults and children through the Influenza Hospitalization Surveillance Network (FluSurv-Net),¶¶ which covers approximately 27 million persons, 8.5% of the U.S. population. From October 1 through December 7, 2013 (week 49), 531 laboratory-confirmed influenza-associated hospitalizations were reported. This yields a rate of 2.0 per 100,000 population. Among cases, 470 (88.5%) were influenza A, 52 (9.8%) were influenza B, four (0.8%) were influenza A and influenza B coinfections, and five (0.9%) had no virus type information. Among those with influenza A subtype information, six (4.1%) were influenza A (H3), and 141 (95.9%) were pH1N1. The most commonly reported chronic underlying medical conditions among adults were obesity, metabolic disorders, chronic lung disease (excluding asthma), and cardiovascular disease. Approximately 42.9% of hospitalized children had no identified chronic underlying medical conditions. The most commonly reported chronic underlying medical conditions in children (those aged <18 years) were asthma, obesity, and metabolic disorders. Among 21 hospitalized women of childbearing age (15–44 years), three were pregnant.

## Pneumonia and Influenza-Associated Mortality

During the week ending December 7, 2013 (week 49), pneumonia and influenza (P&I) was reported as an underlying or contributing cause of 6.2% (791 of 12,758) of all deaths reported to the 122 Cities Mortality Reporting System. This percentage is below the epidemic threshold of 6.8% for that week.*** Since September 29, 2013, the weekly percentage of deaths attributed to P&I ranged from 5.3% to 6.2% and has not exceeded the epidemic threshold so far this season. Peak weekly percentages of deaths attributable to P&I in the previous five seasons ranged from 7.9% during the 2008–09 and 2011–12 seasons to 9.9% during the 2012–13 season.

## Influenza-Associated Pediatric Mortality

As of December 7, 2013 (week 49), three influenza-associated pediatric deaths that occurred in the 2013–14 season were reported to CDC: one death was associated with an influenza A and influenza B virus coinfection, one was associated with an influenza A virus for which no subtyping was performed, and one was associated with a pH1N1 virus. The number of influenza-associated pediatric deaths reported to CDC in the previous three influenza seasons has ranged from 35 during the 2011–12 season to 169 for the 2012–13 season. During the 2009 pandemic, 348 pediatric deaths were reported from April 15, 2009, through October 2, 2010 (traditional influenza seasons include data from October (week 40) through September (week 39) of the following year). In the two seasons before the 2009 pandemic, influenza-associated pediatric deaths reported to CDC ranged from 67 during the 2008–09 season (through April 14, 2009) to 88 during the 2007–08 season.

What is already known on this topic?

CDC collects, compiles, and analyzes data on influenza activity year-round in the United States. The influenza season generally begins in the fall and continues through the winter and spring months; however, the timing and severity of circulating influenza viruses can vary by geographic location and season.

What is added by this report?

During September 29–December 7, 2013, influenza activity overall in the United States has been increasing. The vast majority of the influenza viruses characterized thus far this season have been antigenically like the components included in the 2013–14 Northern Hemisphere trivalent influenza vaccine: A/California/7/2009-like (H1N1), A/Texas/50/2012-like (H3N2), and B/Massachusetts/2/2012-like. The majority of influenza viruses tested to date have been sensitive to the antiviral drug oseltamivir; all are sensitive to the antiviral drug zanamivir.

What are the implications for public health practice?

Vaccination remains the most effective method to prevent influenza and its complications. Health-care providers should offer vaccine to all unvaccinated persons aged ≥6 months now and throughout the influenza season. Treatment with influenza antiviral medications can reduce severe outcomes of influenza, especially when initiated as early as possible, in patients with confirmed or suspected influenza.

### Editorial Note

Influenza activity so far this season has increased during the most recent weeks and is expected to continue to increase in the coming weeks. During September 29–December 7, 2013, pH1N1 viruses were identified most frequently in the United States, but influenza A (H3N2) and influenza B viruses also were reported. Antigenic characterization of influenza-positive respiratory specimens submitted to CDC indicates that 1) the majority of these isolates tested were antigenically like the components of the 2013–14 Northern Hemisphere trivalent influenza vaccine viruses and 2) all of these isolates tested were antigenically like the components of the 2013–14 Northern Hemisphere quadrivalent influenza vaccine viruses. Although the timing of influenza activity varies from year to year, peak activity in the United States most commonly occurs in February, but there can be substantial influenza activity as early as November and December, and activity can occur as late as May (2). Vaccination remains the most effective method to prevent influenza and its complications. Health-care providers should offer vaccine to all unvaccinated persons aged ≥6 months now and throughout the influenza season.

Antiviral medications continue to be an important adjunct to vaccination for reducing the health impact of influenza. On January 21, 2011, Advisory Committee on Immunization Practices recommendations on the use of antiviral agents for treatment and chemoprophylaxis of influenza were released (3). This guidance remains in effect for the 2013–14 season. Treatment with antivirals is recommended as soon as possible for patients with confirmed or suspected influenza who have severe, complicated, or progressive illness; who require hospitalization; or who are at higher risk for influenza complications,††† without waiting for confirmatory testing (3). Antiviral treatment also may be considered for outpatients with confirmed or suspected influenza who do not have known risk factors for severe illness, and is most effective when treatment is initiated within 48 hours of illness onset. Recommended antiviral medications include oseltamivir (Tamiflu) and zanamivir (Relenza). The majority of influenza viruses tested for the 2013–14 season since October 1, 2013, have been susceptible to oseltamivir. The seven oseltamivir-resistant pH1N1 viruses identified in late October through November were from three different states in the South and West. Sporadic oseltamivir-resistant pH1N1 virus infections, including small geographic clusters, have occurred previously, but the public health impact has been limited (4,5). This situation is being closely monitored. All influenza viruses, including those from the oseltamivir-resistant cluster, tested since October 1, 2013, have been susceptible to zanamivir. Amantadine and rimantadine are not recommended because of high levels of resistance to these drugs among circulating influenza A viruses (3). In addition, influenza B viruses are not susceptible to amantadine or rimantadine.

Influenza surveillance reports for the United States are posted online weekly and are available at http://www.cdc.gov/flu/weekly. Additional information regarding influenza viruses, influenza surveillance, influenza vaccine, influenza antiviral medications, and novel influenza A virus infections in humans is available at http://www.cdc.gov/flu.

## Footnotes

* The CDC influenza surveillance system collects five categories of information from eight data sources: 1) viral surveillance (World Health Organization collaborating laboratories, the National Respiratory and Enteric Virus Surveillance System, and novel influenza A virus case reporting); 2) outpatient illness surveillance (U.S. Outpatient Influenza-Like Illness Surveillance Network); 3) mortality (122 Cities Mortality Reporting System and influenza-associated pediatric mortality reports); 4) hospitalizations (Influenza Hospitalization Surveillance Network [FluSurv-NET], which includes the Emerging Infections Program and surveillance in three additional states); and 5) summary of the geographic spread of influenza (state and territorial epidemiologist reports).

† Data reported as of December 13, 2013.

§ *Region 1:* Connecticut, Maine, Massachusetts, New Hampshire, Rhode Island, and Vermont. *Region 2:* New Jersey, New York, Puerto Rico, and the U.S. Virgin Islands. *Region 3:* Delaware, District of Columbia, Maryland, Pennsylvania, Virginia, and West Virginia. *Region 4:* Alabama, Florida, Georgia, Kentucky, Mississippi, North Carolina, South Carolina, and Tennessee. *Region 5:* Illinois, Indiana, Michigan, Minnesota, Ohio, and Wisconsin. *Region 6:* Arkansas, Louisiana, New Mexico, Oklahoma, and Texas. *Region 7:* Iowa, Kansas, Missouri, and Nebraska. *Region 8:* Colorado, Montana, North Dakota, South Dakota, Utah, and Wyoming. *Region 9:* Arizona, California, Hawaii, Nevada, American Samoa, Commonwealth of the Northern Mariana Islands, Federated States of Micronesia, Guam, Marshall Islands, and Republic of Palau. *Region 10:* Alaska, Idaho, Oregon, and Washington.

¶ Defined as a temperature ≥100°F (≥37.8°C), oral or equivalent, and cough and/or sore throat, without a known cause other than influenza.

** The national and regional baselines are the mean percentage of visits for ILI during noninfluenza weeks for the previous three seasons plus two standard deviations. A noninfluenza week is defined as periods of ≥2 consecutive weeks in which each week accounted for <2% of the season’s total number of specimens that tested positive for influenza. National and regional percentages of patient visits for ILI are weighted on the basis of state population. Use of the national baseline for regional data is not appropriate.

†† Activity levels are based on the percentage of outpatient visits in a jurisdiction attributed to ILI and are compared with the average percentage of ILI visits that occur during weeks with little or no influenza virus circulation. Activity levels range from minimal, which would correspond to ILI activity from outpatient clinics being at or below the average, to high, which would correspond to ILI activity from outpatient clinics being much higher than the average. Because the clinical definition of ILI is very nonspecific, not all ILI is caused by influenza; however, when combined with laboratory data, the information on ILI activity provides a clearer picture of influenza activity in the United States.

§§ Levels of activity are 1) no activity; 2) sporadic: isolated laboratory-confirmed influenza case(s) or a laboratory-confirmed outbreak in one institution, with no increase in activity; 3) local: increased ILI, or at least two institutional outbreaks (ILI or laboratory-confirmed influenza) in one region of the state, with recent laboratory evidence of influenza in that region and virus activity no greater than sporadic in other regions; 4) regional: increased ILI activity or institutional outbreaks (ILI or laboratory-confirmed influenza) in at least two but less than half of the regions in the state with recent laboratory evidence of influenza in those regions; and 5) widespread: increased ILI activity or institutional outbreaks (ILI or laboratory-confirmed influenza) in at least half the regions in the state, with recent laboratory evidence of influenza in the state.

¶¶ FluSurv-NET conducts population-based surveillance for laboratory-confirmed influenza-associated hospitalizations among children aged <18 years (since the 2003–04 influenza season) and adults aged ≥18 years (since the 2005–06 influenza season). FluSurv-NET covers approximately 70 counties in the 10 Emerging Infections Program states (California, Colorado, Connecticut, Georgia, Maryland, Minnesota, New Mexico, New York, Oregon, and Tennessee) and additional Influenza Hospitalization Surveillance Project (IHSP) states. IHSP began during the 2009–10 season to enhance surveillance during the 2009 H1N1 pandemic. IHSP sites included Iowa, Idaho, Michigan, Oklahoma, and South Dakota during the 2009–10 season; Idaho, Michigan, Ohio, Oklahoma, Rhode Island, and Utah during the 2010–11 season; Michigan, Ohio, Rhode Island, and Utah during the 2011–12 season; Iowa, Michigan, Ohio, Rhode Island, and Utah during the 2012–13 season; and Michigan, Ohio, and Utah during the 2013–14 season. Incidence rates are calculated using CDC’s National Center for Health Statistics population estimates for the counties included in the surveillance catchment area. Laboratory confirmation is dependent on clinician-ordered influenza testing, and testing for influenza often is underutilized because of the poor reliability of rapid test results and greater reliance on clinical diagnosis for influenza. As a consequence, the number of cases identified as part of influenza hospitalization surveillance likely is an underestimation of the actual number of persons hospitalized with influenza.

*** The seasonal baseline proportion of P&I deaths is projected using a robust regression procedure, in which a periodic regression model is applied to the observed percentage of deaths from P&I that were reported by the 122 Cities Mortality Reporting System during the preceding 5 years. The epidemic threshold is set at 1.645 standard deviations above the seasonal baseline.

††† Persons at higher risk include 1) children aged <5 years (especially those aged <2 years); 2) adults aged ≥65 years; 3) persons with chronic pulmonary conditions (including asthma); cardiovascular disease (except hypertension alone); renal, hepatic, or hematologic (including sickle cell) disease; metabolic disorders (including diabetes mellitus); or neurologic and neurodevelopment conditions (including disorders of the brain, spinal cord, peripheral nerve, and muscle, such as cerebral palsy, epilepsy [seizure disorders], stroke, intellectual disability [mental retardation], moderate to severe developmental delay, muscular dystrophy, or spinal cord injury); 4) persons with immunosuppression, including that caused by medications or by human immunodeficiency virus infection; 5) women who are pregnant or postpartum (within 2 weeks after delivery); 6) persons aged ≤18 years who are receiving long-term aspirin therapy; 7) American Indians/Alaska Natives; 8) persons who are morbidly obese (i.e., body mass index ≥40); and 9) residents of nursing homes and other chronic-care facilities.
